# Contraction of axonal and dendritic fields in *Sox5*-deficient cone bipolar cells is accompanied by axonal sprouting and dendritic hyper-innervation of pedicles

**DOI:** 10.3389/fnana.2022.944706

**Published:** 2022-08-19

**Authors:** Bridget Kulesh, Benjamin E. Reese, Patrick W. Keeley

**Affiliations:** ^1^Neuroscience Research Institute, University of California, Santa Barbara, Santa Barbara, CA, United States; ^2^Department of Molecular, Cellular and Developmental Biology, University of California, Santa Barbara, Santa Barbara, CA, United States; ^3^Department of Psychological and Brain Sciences, University of California, Santa Barbara, Santa Barbara, CA, United States

**Keywords:** outer plexiform layer, inner plexiform layer, cone pedicle, convergence, stratification, conditional knockout

## Abstract

Multiple factors regulate the differentiation of neuronal morphology during development, including interactions with afferents, targets, and homotypic neighbors, as well as cell-intrinsic transcriptional regulation. Retinal bipolar cells provide an exemplary model system for studying the control of these processes, as there are 15 transcriptionally and morphologically distinct types, each extending their dendritic and axonal arbors in respective strata within the synaptic layers of the retina. Here we have examined the role of the transcription factor *Sox5* in the control of the morphological differentiation of one type of cone bipolar cell (CBC), the Type 7 cell. We confirm selective expression of SOX5 in this single bipolar cell type, emerging at the close of the first post-natal week, prior to morphological differentiation. Conditional knockout mice were generated by crossing a bipolar cell-specific *cre*-expressing line with mice carrying floxed *Sox5* alleles, as well as the *Gustducin-gfp* reporter which labels Type 7 CBCs. Loss of SOX5 was confirmed in the bipolar cell stratum, in GFP+ Type 7 cells. Such SOX5-deficient Type 7 cells differentiate axonal and dendritic arbors that are each reduced in areal extent. The axonal arbors exhibit sprouting in the inner plexiform layer (IPL), thereby extending their overall radial extent, while the dendritic arbors connect with fewer cone pedicles in the outer plexiform layer, showing an increase in the average number of dendritic contacts at each pedicle. SOX5-deficient Type 7 CBCs should therefore exhibit smaller receptive fields derived from fewer if now hyper-innervated pedicles, transmitting their signals across a broader depth through the IPL.

## Introduction

The processes that contribute to the acquisition of neuronal morphology during development are multifarious. The distribution and density of afferents influence the dendritic differentiation of a neuronal population, while axonal arbors are modulated by the population of target cells they seek to innervate (Gibson and Ma, 2011; Lin et al., 2020). These afferents and targets may play a synaptotropic role in guiding process outgrowth, or they may play a neurotrophic role in promoting differentiation (McAllister, 2001; Cline and Haas, 2008). The local density of neighboring cells within the population may also affect dendritic as well as axonal differentiation, serving as a competing source for those afferents or targets, respectively, or by constraining outgrowth directly through homotypic interactions (Parrish et al., 2007; Inberg et al., 2019). Intrinsic factors additionally play a role in the morphological differentiation of distinct types of neurons, by directing the cell to form certain aspects of its differentiated state cell autonomously (Dunn et al., 1998; Wu et al., 2015), or by ensuring that a particular type of cell is sensitive to cues provided by others in the local environment (Luo et al., 2016; Peng et al., 2017). Together, these cell-intrinsic and intercellular dependencies sculpt the unique morphologies of mature neuronal cell types.

The retina is an attractive model system for studying the factors controlling the acquisition of neuronal morphology, as the different neuronal populations are situated in discrete cellular layers, with their dendritic and axonal processes commonly stratified within dedicated synaptic, or plexiform, layers. Indeed, the retinal bipolar cell is an ideal cell type for exploring the control of neuronal differentiation, as there are multiple types of retinal bipolar cells, each with its own characteristic dendritic morphology in the outer plexiform layer (OPL), where they receive synaptic input from photoreceptors, and with a unique pattern of axonal termination within the inner plexiform layer (IPL), where these cells form synaptic contacts with distinct types of amacrine and ganglion cells. The mouse retina, for instance, contains 14 different types of cone bipolar cell (CBC) and 1 type of rod bipolar cell (RBC), each varying in the spread, stratification, and connectivity of its dendritic and axonal arbors (Helmstaedter et al., 2013; Behrens et al., 2016; Shekhar et al., 2016).

The present investigation has explored the role of the transcription factor *Sox5* in the control of bipolar cell differentiation. *Sox5* is a member of the *Sry*-related HMG box gene family, encoding a diverse number of well-conserved transcription factors (Pevny and Placzek, 2005; Stevanovic et al., 2021), but a role for *Sox5* in the development of the nervous system has gone relatively unexplored. It has been shown to regulate the morphological differentiation of select neuronal populations in mice and *Drosophila* (Kwan et al., 2008; Li et al., 2017), and has recently been shown to be expressed in one type of cone bipolar cell in the mouse retina (Shekhar et al., 2016), the Type 7 CBC. Here we used a genetic strategy to eliminate *Sox5* function in this bipolar cell type in order to explore its contribution to neuronal differentiation *in vivo*.

## Materials and Methods

### Mice

The following mouse strains were obtained from the Jackson Laboratory: 129S1.Cg-Tg(Vsx2-cre)2690Chow/J (RRID:IMSR_JAX:026200) and Gt(ROSA)26Sortm9(CAG-tdTomato)Hze/J (RRID:IMSR_JAX:007909; *Ai9* hereafter). We also used the Tg(Gnat3-GFP)1Rfm/ChowJ (RRID:IMSR_JAX:026704; *Gustducin*-*gfp* hereafter) reporter mouse (Huang et al., 2003), originally obtained from the laboratory of Richard Masland, and currently available from the Jackson Laboratory. Sox5tm2Vlf mice (RRID:MGI:3800352), carrying a floxed allele of Sox5, were obtained from the laboratory of Véronique Lefebvre at The Cleveland Clinic (Dy et al., 2008). These lines were bred and then crossed to yield *Sox5*-conditional knockout (CKO) mice additionally reporting Cre activation as well as selectively identifying Type 7 CBCs.

### Tissue preparation

Mice (4–8 weeks of age) were given a lethal injection of sodium pentobarbital (120 mg/kg) and once deeply anesthetized, were perfused intracardially using 2–3 ml physiological saline followed by ~75 ml 4% paraformaldehyde in 0.1 M sodium phosphate buffer (PB; pH 7.2–7.4), delivered by gravity over 15 min. Eyes were dissected and immersed in fixative for a further 15 min. For DiI injections, eyes were immediately removed from deeply anesthetized mice and placed in fixative for 5 min, at which point the cornea and lens were removed, and eyecups returned to fixative for an additional 25 min.

**Figure 1:**
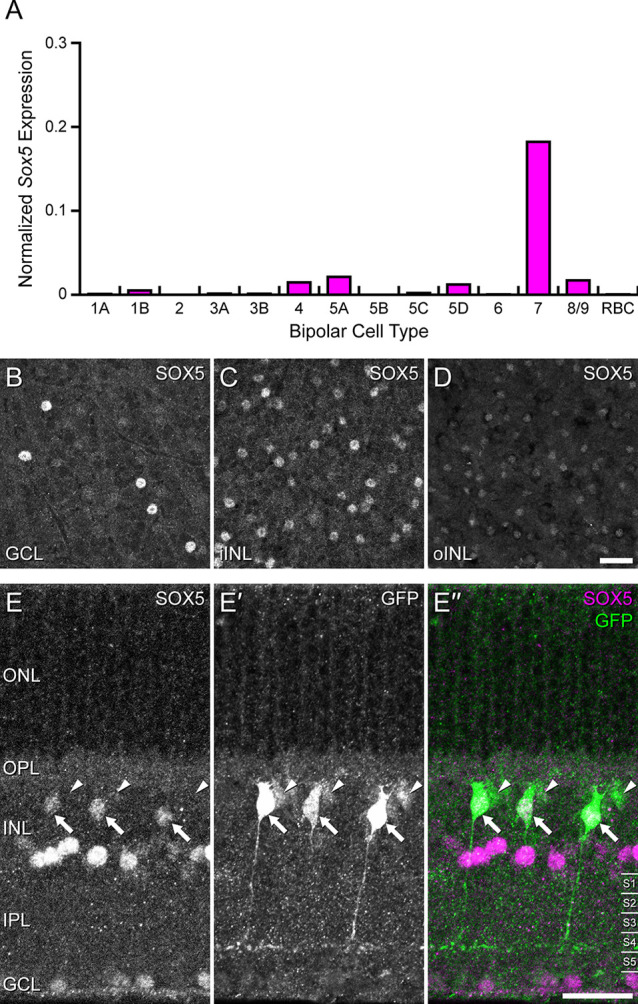
**(A)** Single cell transcriptional profiling of retinal bipolar cells demonstrated that *Sox5* expression is near-selectively expressed in only one the Type CBC, the Type 7 CBC (from Shekhar et al., 2016; accessed from the single cell portal from the Broad Institute: https://singlecell.broadinstitute.org/single_cell). For each cell, the number of transcripts for each gene was adjusted to account for sequencing depth, and then log transformed, ln(x + 1), as described in Shekhar et al. (2016). Each histogram plots the average of this normalized expression across all cells for each bipolar cell type. **(B–D)** Immunostaining of mature mouse retinal wholemounts using an antibody to SOX5 reveals labeled cells in the GCL **(B)** and in the INL, the latter including cells in the innermost portion (iINL), being the amacrine cell layer **(C)**, as well as cells in the outermost portion (oINL), being the bipolar cell stratum **(D)**. **(E–E”)** Immunostaining *Gustducin-gfp* transgenic retinas for SOX5 **(E–E”)** confirms that the SOX5-positive cells in the bipolar cell stratum are also GFP+ (**E’**, arrows). These are confirmed to be Type 7 CBCs by virtue of their stratifying axonal arbors in S4 of the IPL. Note three GFP-positive cells that do not co-localize SOX5 in their somata (arrowheads in **E–E”**); these are RBCs, evidenced by their dimmer GFP fluorescence and by their axons reaching into S5 of the IPL. Calibration bars = 50 μm.

Retinas were subsequently dissected from the eyecups, and then either prepared as retinal wholemounts or embedded flat in 5% agarose in 0.1 M PB for sectioning. Radial sections through the retina were cut at 200 μm using a PELCO easySlicer (Ted Pella, Inc., Redding, CA). Wholemounts or sections were immunostained according to the following protocol: Tissues were incubated in 5% normal donkey serum for 3 h, followed by a series of three washes in cold phosphate-buffered saline (PBS). Tissues were then incubated in primary antibodies, for 3 days. After an additional three washes in cold PBS, tissues were incubated overnight with secondary antibodies and then washed three times in PBS. All incubations were done at 4°C with gentle agitation, and all solutions were made up of 1% Triton X-100 in PBS. Hoechst 33342 (Invitrogen, #H3570) was used at a dilution of 1:1,000. A rabbit polyclonal antibody to SOX5 was used to determine expression patterns in developmental and adult tissue (Thermofisher Scientific, #PA5-66331, 1:500; RRID:AB_2662817). This antibody was raised against a 92aa-long peptide corresponding to the human SOX5 protein (aa39-aa130), a region which is over 90% homologous to mouse SOX5. We tested three dilutions of this antibody to determine a suitable working concentration in the retina and included a negative control to assess any background staining caused by the secondary antibodies. Positive staining (e.g., Figure 1) by the primary antibody was localized to cell nuclei in the GCL and INL, but not ONL, which is consistent with the role of SOX5 as a transcription factor and with predicted expression patterns based on single cell RNA seq atlases of the mouse retina (Shekhar et al., 2016; Tran et al., 2019; Yan et al., 2020; accessible from the Single Cell Portal at the Broad Institute^1^). Critically for the validation of this primary antibody, SOX5 labeling was ablated in conditional knockout mice as expected (shown in results). Two antibodies were used in order to detect and amplify the GFP signal: a rabbit polyclonal antibody conjugated to AlexaFluor488 (Thermofisher Scientific, #A21311, 1:1,000; RRID:AB_221477) and a chicken polyclonal antibody (Thermofisher Scientific, #A10262, 1:1,000; RRID:AB_2534023). An affinity-purified rabbit polyclonal antibody to Cone Arrestin (1:10,000, Millipore, #AB15282; RRID:AB_1163387) and peanut agglutinin lectin (PNA) conjugated to AlexaFluor647 (1:200; Thermofisher Scientific, L32460) were used to identify cones. Secondary antibodies raised in donkey and directed to either rabbit IgG and conjugated to AlexaFluor488 (1:200, Invitrogen, #A21206; RRID:AB_2535792) or AlexaFluor546 (1:200, Invitrogen, #A10040; RRID:AB_2534016), or chicken IgY conjugated to AlexaFluor488 (1:200, Jackson ImmunoResearch, 703-545-155; RRID:AB_2340375) were used to detect the primary antibodies.

### DiI injections

Retinal wholemounts were placed in a Petri-dish with the ganglion cell layer up and held in place with a piece of weighing paper, itself held down by magnets, in which a small window had been cut to reveal all but the peripheral portions of each retinal quadrant. The dish was filled with 0.1 M PB and transferred to a fixed-stage Nikon microscope equipped with a Burleigh micromanipulator. A small glass capillary was pulled into a pipette, with a tip diameter of approximately 0.5 microns; this pipette was backfilled with a solution of CM-DiI (Invitrogen, V22888). The tip of the pipette was guided to either GFP-positive axon terminals in the IPL (to label dendritic arbors) or to GFP-positive somata in the INL (to label axonal arbors), and a small deposit of DiI was expelled by passing a positive current through the pipette for several seconds. Once each quadrant of the retina was filled with a dozen or so of these injections, retinas were immersion-fixed for an additional hour, rinsed three times with PBS, then left overnight in a solution of PNA conjugated to AlexaFluor647. The next day, retinas were rinsed and mounted on glass slides, and individual dendritic or axonal arbors were imaged using an Olympus Fluoview 1,000 laser scanning confocal microscope equipped with a ×40 oil-immersion objective with a numerical aperture of 1.30. For each DiI-labeled dendritic and axonal arbor that was imaged, the native GFP signal was also imaged at the level of the soma and of the axonal stalk, respectively, to confirm the identity of the injected cell as either a Type 7 CBC or an RBC; the clear morphological differences between the two cell types made identifying the Type 7 CBCs unambiguous, as described previously (Keeley and Reese, 2010).

### Morphometric analysis and statistics

Labeled axonal and dendritic arbors of DiI-labeled Type 7 CBCs were sampled in retinal wholemounts through their entire depth within the IPL and OPL, respectively. Axonal arbor depth (specifically, the extent of the arbor across the depth of the IPL) was quantified by counting the number of 0.5 μm optical sections encompassing labeled processes for each cell. Z-stack projections of the entire axonal and dendritic arbors were respectively generated, and a convex polygon was drawn around each arbor to estimate its areal extent. Somal perimeters were traced to determine their areas, as were the retinal wholemounts. Cone numbers were compared by sampling the local density of cone pedicles labeled with PNA and antibodies to Cone Arrestin. Fields 0.011 mm^2^ in area were sampled using a ×40 objective, taken from both a central and a peripheral location in each quadrant of the retinal wholemount. Density was determined from the eight fields to generate an average for each retina.

The number of cones contacted by each labeled dendritic arbor was determined by the colocalization of DiI-labeled dendritic tips at PNA-labeled cone pedicles. The theoretical point spread function was calculated for each of the images^2^, and images were then de-convolved^3^ in order to enhance the dendritic tips at each pedicle. All of the above quantifications of filled cells, cone densities, and retinal areas were conducted blind to condition, with filled cells and cone sample fields additionally being coded and then randomly intermingled prior to quantification. Student’s t-tests were used to determine significant differences between the populations derived from *Sox5*-CKO and littermate control mice, using a *p-*value of 0.05.

## Results

Transcriptional profiling of single retinal neurons in maturity has demonstrated *Sox5* expression in a minority of amacrine cell types and in one type of retinal ganglion cell (Tran et al., 2019; Yan et al., 2020). *Sox5* was also shown to be expressed primarily in one type of CBC, the Type 7 cell (Shekhar et al., 2016; Figure 1A). Immunolabeling mature retina confirmed SOX5 expression in cells in the ganglion cell layer (GCL; Figure 1B), in the inner parts of the INL (iINL, being the amacrine cell layer; Figure 1C), and in the outer parts of the INL (oINL), where bipolar cells are positioned (Figure 1D). These SOX5 immuno-positive cells in this bipolar cell stratum were shown to be Type 7 CBCs by virtue of their also expressing the *Gustducin-gfp* reporter (arrows in Figures 1E–E’’), selectively expressed in Type 7 CBCs and RBCs (Huang et al., 2003). Those that co-express GFP and SOX5 in cross-sections of the retina can be confirmed to be Type 7 CBCs by virtue of their axonal stratification in S4 of the IPL (Figure 1E’), rather than S5, where rod bipolar axons terminate.

**Figure 2:**
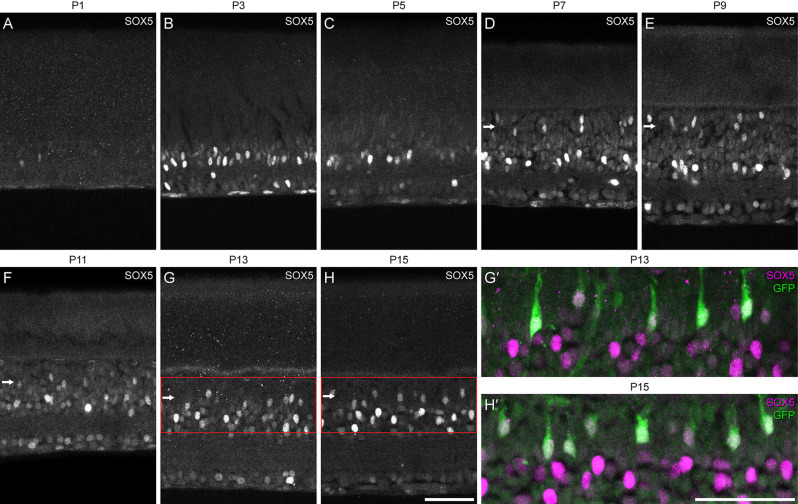
**(A–H)** Developmental time-course of the emergence of SOX5-immunolabeling in the retina, from the day of birth through the first two postnatal weeks. Note the onset of labeling in cells in the emerging bipolar cell stratum at the close of the first postnatal week **(D)**, remaining thereafter (horizontal arrows). Panels **(G’,H’)** show magnified regions of the INL from panels **(G)** and **(H)**, confirming that those SOX5-immunopositive cells in the bipolar cell stratum (magenta) are Type 7 CBCs, as they are also GFP-positive (green). Calibration bars = 50 μm.

We first defined the developmental time course of SOX5 expression revealed *via* immunofluorescence to confirm its presence during retinal development. On the day of birth, hardly any cells were SOX5-immunopositive (Figure 2A), but beginning at P3, a minority of cells in the emerging amacrine cell layer in the developing INL and in the GCL became strongly immunopositive (Figure 2B). Beginning around P7, an additional population of SOX5 cells in the INL became immunopositive, being situated in the outer portion of the INL, the emerging bipolar cell stratum (arrows in Figures 2D–H). These were confirmed to be Type 7 CBCs at the later ages of P13 and P15, in retinas expressing the *Gustducin-gfp* reporter (Figures 2G’,H’).

**Figure 3:**
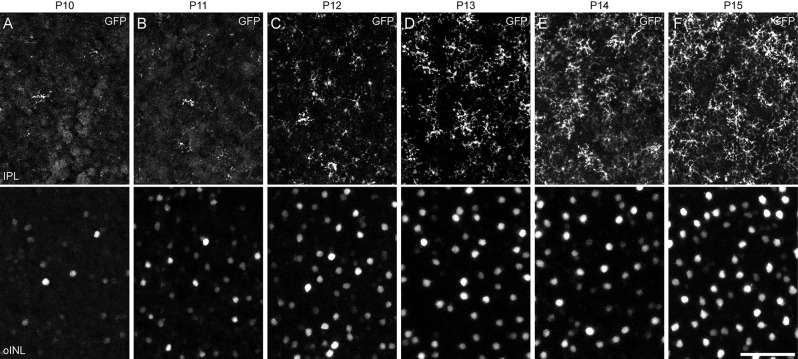
**(A–F)** Developmental time-course of Type 7 CBC differentiation, revealed through GFP-labeling of their differentiating axon terminals in S4 of the IPL (top row) and their respective somata in the INL (bottom row). Axonal arbors in S4 are first detected at P10 **(A)**, increasing in frequency over the following 5 days **(B–F)**, when they are also increasing the size of their territories. As *Gustducin-gfp* is incompletely expressed by the population of Type 7 cells, there remain occasional gaps within the mosaic of axon terminal arbors at P15, as in maturity. Calibration bar = 50 μm.

We compared this onset of SOX5 expression with the morphological differentiation of Type 7 CBCs, revealed by the GFP labeling of Type 7 CBCs during development. Type 7 CBC axon terminals were first detected sporadically in the IPL on P10. They became increasingly frequent during the remainder of the second postnatal week (Figure 3, top row), when the frequency of their GFP-positive somata increased (Figure 3, bottom row). During this period, their axon terminals increased their arboreal spread as they matured. While we were unable to observe the progression of dendritic morphology during this time period using the *Gustducin-gfp* mouse, a previous study of developing ON CBCs showed initial outgrowth of dendritic processes begins only at the end of the first postnatal week (Morgan et al., 2006). As the Type 7 CBC is one of the last bipolar cell types generated (West et al., 2022), those earliest differentiating dendritic arbors are unlikely to arise from the Type 7 cell. Expression of *Sox5* in Type 7 CBCs, consequently, precedes their morphological differentiation.

**Figure 4:**
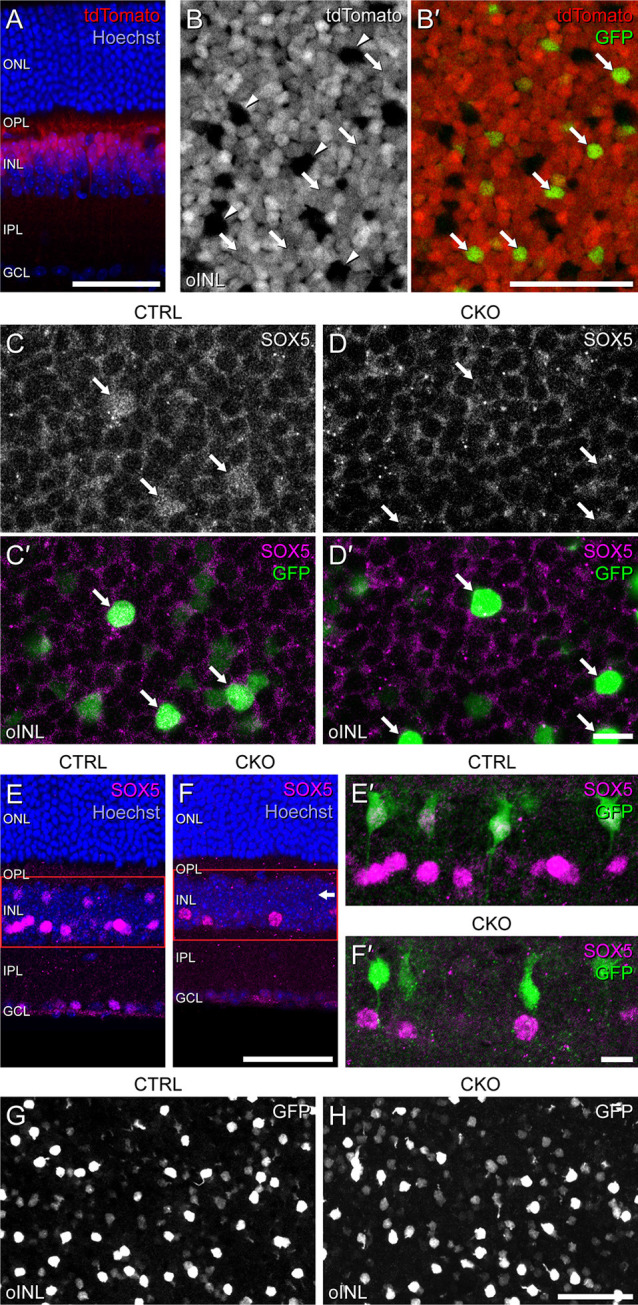
*Sox5*-CKO mice were bred to harbor floxed alleles of *Sox5*, the *Vsx2-cre* transgene, the *Ai9 cre-*reporter transgene, and the *Gustducin-gfp* reporter transgene. **(A)** Retinal cross-section from a mature littermate control mouse revealing the laminar restriction of the tdTomato labeling, confined to the outer half of the INL where bipolar cells are situated. **(B,B’)** Wholemounted retina from a mature littermate control mouse through the bipolar cell stratum of the INL, showing most cells to be tdTomato-positive. Arrowheads indicate a minority of cells that are tdTomato-negative **(B)**, including the population of horizontal cells, while arrows indicate a minority of tdTomato-positive cells that are also GFP-positive **(B’)**, confirming that these are Type 7 CBCs. **(C,C’,D,D’)** In *Sox5*-CKO retinas, the bright GFP+ cells (arrows) are no longer SOX5-immunopositive **(D,D’)**, shown in retinal wholemounts. **(E,E’,F,F’)** *Sox5*-CKO retinas show a loss of SOX5 labeling in the bipolar cell stratum of the INL (horizontal arrow), shown here in section **(E,F)**. Higher magnification images of the INL in the same sections reveal GFP-positive cells remain present but are not SOX5-positive in the *Sox5*-CKO retinas **(E’,F’)**. **(G,H)** Despite this absence of SOX5-positive cells, the population of GFP-positive cells is not diminished in *Sox5*-CKO retinas, enabling them to be targeted for DiI injection. Calibration bars = 50 μm in panels **(A,B,B’,E,F,G,H)** and = 10 μm in panels **(C,C’,D,D’,E’,F’)**.

Conditional deletion of *Sox5* was accomplished by crossing genetically modified lines of mice to yield targeted offspring with floxed alleles of *Sox5* along with the *Vsx2-5.3-PRE-cre* transgene (abbreviated *Vsx2-cre* hereafter), shown to drive expression of Cre exclusively in postmitotic bipolar cells, evidenced by its restriction to the bipolar cell stratum (Nickerson et al., 2011). Activation of *cre* in these mice has been shown to commence around P3, becoming widespread within the bipolar cell population by P6 (Nickerson et al., 2011). To confirm that *cre* is expressed within Type 7 CBCs, we bred the *Ai9 cre-*reporter (expressing tdTomato in positive cells) as well as the *Gustducin-gfp* reporter (Wong et al., 1999) onto these mice. Sections of the retina confirmed widespread tdTomato labeling exclusively within the bipolar cell stratum of the INL (Figure 4A). In wholemount preparations, this tdTomato labeling excluded the horizontal cells, notable by their larger somal sizes (Figure 4B, arrowheads). Critically, those bipolar cells that were GFP-positive were also tdTomato-positive, indicating Cre to be active within Type 7 CBCs (Figures 4B,B’, arrows). As expected, in *Sox5*-CKO retinas, Cre-mediated recombination eliminated *Sox5* in these cells, confirmed by the loss of SOX5 immunolabeling in GFP-positive Type 7 CBCs (Figures 4C,C’,D,D’). While SOX5 labeling was absent from cells in the bipolar cell stratum of the INL in the CKO retinas (Figures 4E,F, arrow), specifically, the GFP-positive cells (Figures 4E’,F’), it remained within those cells positioned in the amacrine cell stratum, confirming the expected specificity of the deletion. Despite the loss of SOX5-positive nuclei in the bipolar cell stratum of *Sox5*-CKO retinas, the incidence of GFP-positive somata was comparable to that found in littermate control retinas (Figures 4G,H).

**Figure 5:**
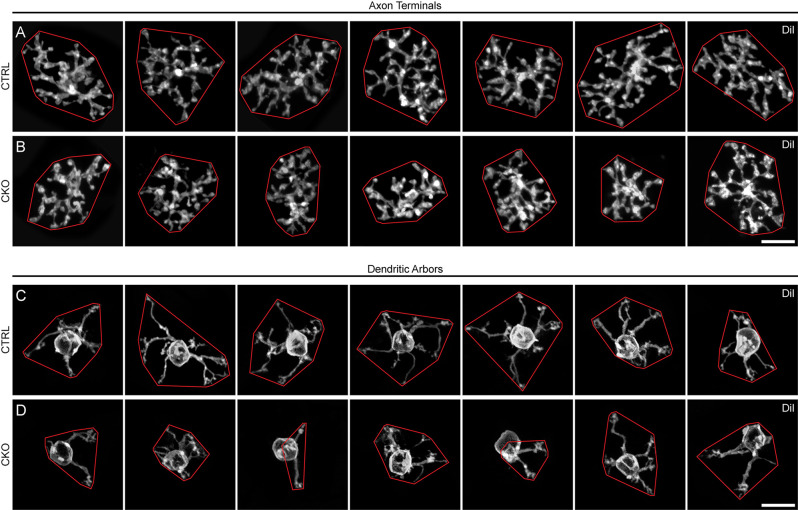
**(A–D)** Seven examples of DiI-labeled axon terminals **(A,B)** and dendritic arbors **(C,D)** from *Sox5*-CKO **(B,D)** and littermate control **(A,C)** retinas, in Z-stack projections from retinal wholemounts. Note the smaller areal domains of both axonal and dendritic territories in the *Sox5*-CKO retinas, enclosed here by convex polygons (red). Calibration bar = 10 μm.

GFP-positive Type 7 CBCs in *Sox5*-CKO mice had morphologies largely characteristic of normal Type 7 cells (Helmstaedter et al., 2013). An axon extended basally from the soma into the IPL to produce a stratified axonal arbor in S4, while a large apically directed dendritic stalk gave rise to multiple secondary dendrites within the OPL. Through breeding these mice, however, we noticed that pups could inherit either the *Gustducin-gfp* transgene or the *Vsx2-cre* transgene from a single parent but never both, indicating that these two transgenes were located on the same chromosome. The consequence of this was that CKO mice, needing the *cre* transgene from one parent, could only inherit a single copy of the *Gustducin-gfp* allele from the other, resulting in the immuno-amplified GFP fluorescence being reduced relative to our previous studies using mice that were homozygous for the reporter (Keeley and Reese, 2010). Consequently, while such GFP labeling of Type 7 CBCs was effective in revealing the qualitative features of the axonal and dendritic arbors, these labeled processes lacked sufficient signal to examine the finer aspects of their morphologies; in particular, it was difficult to discern the details of the axonal terminal arbors within the IPL, and the individual dendritic terminals extending into cone pedicles in the OPL. To better visualize changes in the axonal arbor, we labeled single Type 7 CBCs in wholemounts from *Sox5-*CKO and littermate control retinas by injecting the lipophilic dye, DiI, into the soma, targeted by virtue of its GFP fluorescence (e.g., Figures 4G,H). The detailed dendritic structure was revealed in a separate group of cells by injecting the GFP-labeled axonal arbor, all as detailed elsewhere (Keeley and Reese, 2010). *En face* reconstructions of such axonal and dendritic arbors labeled using DiI displayed morphologies that were qualitatively similar (Figures 5A–D, respectively). Axonal arbors were characteristically stratified in the IPL, while dendritic arbors exhibited the periodic clustering of dendritic endings within the OPL that associate with individual cone pedicles.

**Figure 6:**
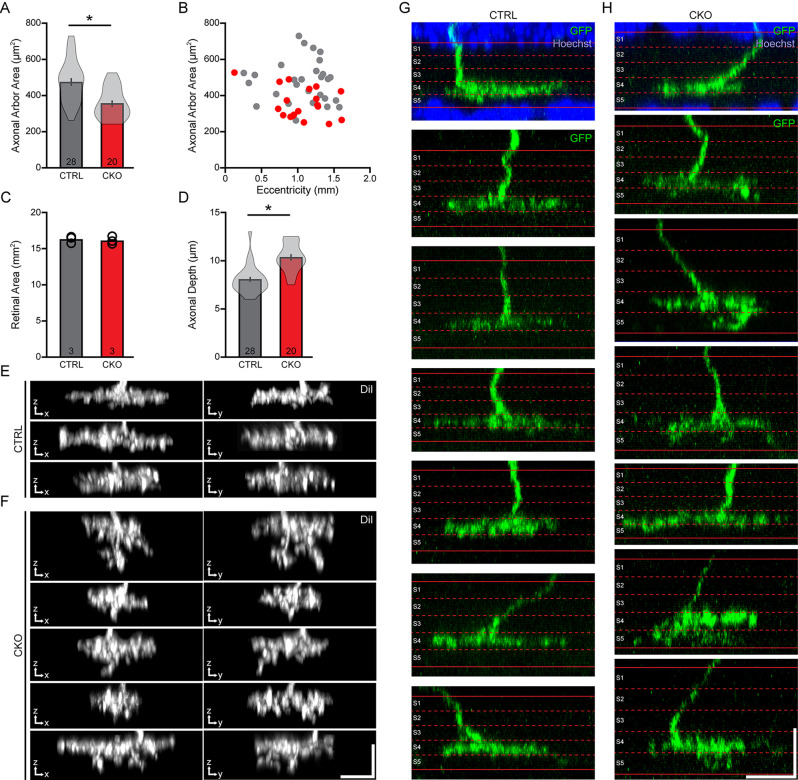
**(A–C)** The areas occupied by the axonal arbors were significantly reduced in the *Sox5*-CKO retinas **(A)**, measured from populations of labeled cells drawn from comparable retinal eccentricities **(B)** and in retinas showing no difference in overall areal size **(C)**. **(D–F)** The stratification of these arbors was significantly broadened across the depth of the IPL **(D)**, largely due to the presence of vertical sprouts descending from the arbor **(E,F)**. Each pair of images shows the X-Z and Y-Z projection of the entire axonal arbor, for multiple cells in each condition. **(G,H)** Positioning of these axonal arbors, relative to the boundaries of the IPL defined by Hoechst staining (shown for two examples), was found to be typical of Type 7 CBCs, in S4 of the IPL, but for the ectopic sprouting extending into S5. Calibration bars = 10 μm. Means and standard errors are shown within the violin plots in **(A,D)**, while means and individual retinas are indicated in **(B)**. n = the number of cells **(A,D)** or retinas **(C)** sampled.

We measured the areal domains occupied by the DiI-labeled axon terminal fields from 28 cells in control retinas and 20 cells in CKO retinas. The axonal arbor area was reduced by an average of 25% in the *Sox5*-CKO retina (Figure 6A; *p* < 0.001). We confirmed that this difference was not associated with any differential sampling of cells across retinal eccentricity between the two conditions (Figure 6B), and nor was it associated with any reduction in total retinal area in the *Sox5-CKO* (Figure 6C; *p* = 0.24). We also assessed the stratification of the axonal arbors by measuring the thickness of these labeled processes (specifically, their presence across sequential optical sections) in the IPL, finding a significant expansion within the IPL by about 29% in the *Sox5*-CKO retinas (Figure 6D; *p* < 0.001). This expansion was largely driven by the presence of ectopic sprouting from an otherwise normally stratifying arbor, extending further vitreally into the IPL (Figures 6E,F). While the limits of the IPL were not determined when sampling the thickness of these DiI-labeled arbors, other GFP-labeled arbors in *Sox5*-CKO retinas were reconstructed relative to the boundaries of the IPL, confirming their normal positioning in S4, but for this additional ectopic sprouting into S5 (Figures 6G,H).

**Figure 7:**
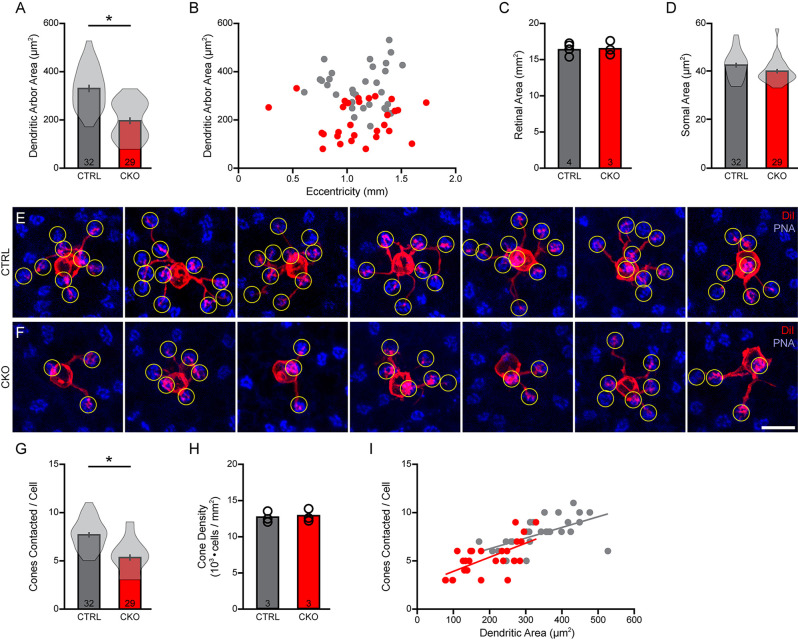
**(A–D)** The areas occupied by the dendritic arbors were also significantly reduced **(A)**, again drawn from samples of cells distributed across similar retinal eccentricities **(B)** from retinas of comparable areas **(C)**. Soma sizes were also determined, revealing a slight but non-significant reduction in somal area **(D)**. **(E,F)** Fields containing DiI-labeled dendritic arbors (red) were also labeled for PNA-positive cone pedicles (blue), in order to quantify the number of pedicles contacted (yellow circles) in *Sox5*-CKO **(F)** and littermate control **(E)** retinas. Calibration bar = 10 μm. **(G)** The number of cone pedicles contacted by those dendritic arbors was significantly reduced in the *Sox5*-CKO retina. **(H)** Average cone densities were unchanged in the *Sox5*-CKO retina. **(I)** The number of cone pedicles contacted increased with the areal size of the dendritic arbor, in both groups. Means and standard errors are shown within the violin plots in **(A,D,G)**, while means and individual retinas are indicated in **(C,H)**. n = the number of cells **(A,D,G)** or retinas **(C,H)** sampled.

A similar trend was found for the areal size of the DiI-labeled dendritic arbors, assessed in 32 cells from control retinas and 29 cells in *Sox5*-CKO retinas. Dendritic field area was reduced by an average of 41% in the absence of *Sox5* (Figure 7A; *p* < 0.001), sampling again populations from comparable eccentricities (Figure 7B), from retinas of similar areal size (Figure 7C; *p* = 0.81). Somal areas were also measured from these cells, showing a slight, if a non-significant reduction in their sizes in the *Sox5-*CKO retinas (Figure 7D; *p* = 0.07).

Those smaller dendritic fields should in principle overlie fewer cone pedicles. These same retinas were each labeled using PNA to reveal the positions of the cone pedicles (Figures 7E,F), from which we quantified the number of pedicles contacted by each labeled Type 7 CBC. There was a significant reduction in the number of cone pedicles contacted, from an average of 7.72 in the littermate control retinas, to 5.34 in the *Sox5*-CKO retinas, being a 31% reduction (Figure 7G; *p* < 0.001). We confirmed that this reduction was not a consequence of any change in the number of cone photoreceptors in these *Sox5*-CKO retinas (Figure 7H; *p* = 0.52). As in control retinas, so in the *Sox5*-CKO retinas, the number of cones contacted was correlated with the size of the dendritic field area (Figure 7I).

**Figure 8:**
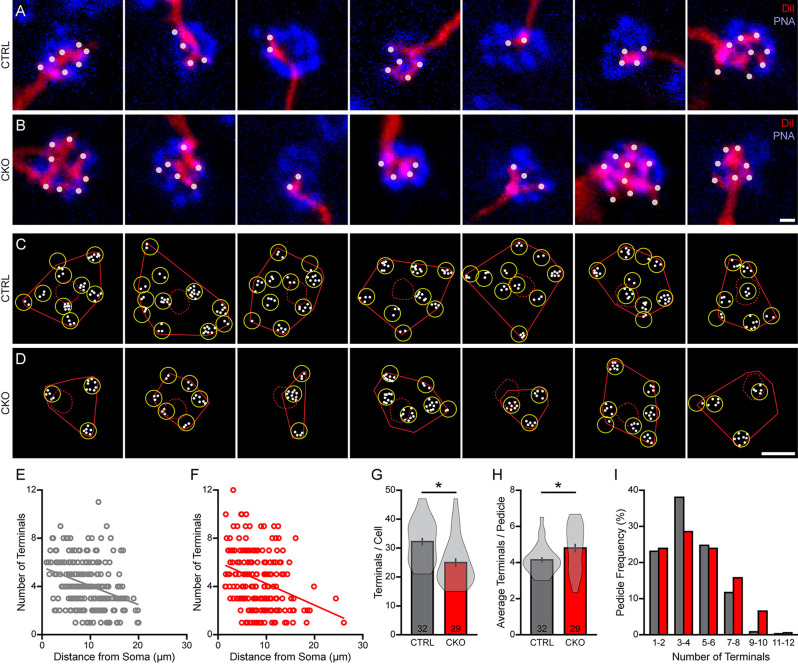
**(A–D)** The number of DiI-labeled dendritic endings (white dots) at individual PNA-labeled cone pedicles (blue) **(A,B)** showed considerable variability across the dendritic fields **(C,D)**, in both littermate control **(A,C)** and in *Sox5-*CKO retinas **(B,D)**. Calibration bar = 1 μm in panels **(A,B)**, and = 10 μm in panels **(C,D)**. **(E,F)** The number of terminals per pedicle declined gradually with distance from the labeled Type 7 soma, in both conditions. **(G,H)** As expected, the total number of dendritic terminals contacting all of the pedicles by a cell was significantly reduced in the *Sox5*-CKO retina **(G)**, but the average number at each pedicle per cell was significantly increased **(H)**. The average number of dendritic terminals at each pedicle per cell is considerably more variable in the *Sox5*-CKO retinas, due primarily to the presence of hyper-innervated pedicles **(H)**. **(I)** Pooling the entire population of sampled pedicles, the frequency distribution of the number of pedicles with a given number of terminals is shifted to the right in the *Sox5*-CKO retinas.

Each bipolar cell dendrite targeting a particular pedicle gives rise to a number of fine dendritic tips that form invaginations into the pedicle at ribbon synapses (Vardi et al., 1998; Dunn and Wong, 2012). These individual terminals can be resolved using DiI and then quantified at each pedicle (Figures 8A,B). Their number per pedicle varied widely across the dendritic field (Figures 8C,D), from as few as one or two terminals to as many as 12, showing a comparable declining trend as a function of distance from the soma (Figures 8E,F; Keeley and Reese, 2010). Nearly all of them were positioned within 20 μm of their somata (Behrens et al., 2016) but the cells in *Sox5*-CKO retinas showed relatively fewer contacted pedicles at the greater distances. There was, not surprisingly, a significant reduction in the total number of these dendritic terminals per cell in the *Sox5-CKO retinas*, by 22% (Figure 8G; *p* < 0.001), to be expected given the fewer pedicles contacted (Figure 7G). Nevertheless, the average number of terminals per pedicle, for each cell, increased in the *Sox5-*CKO retina (Figure 8H; *p* = 0.02), with more of the most densely innervated pedicles present in the *Sox5*-CKO retinas (Figure 8I). Notably, the variance in the average number per cell was considerably greater in the *Sox5*-CKO (Figure 8H), with more cells containing elevated average numbers of terminals per pedicle. Together, these results indicate that cone-to-bipolar cell connectivity is altered in the absence of *Sox5*, with Type 7 CBCs having smaller dendritic fields that now hyper-innervate their pedicles.

## Discussion

The developmental trajectories of the different types of CBC appear to be distinct, differentiating their respective morphologies at different rates, some acquiring their mature dendritic connectivity early while others continue to refine their dendritic connectivity weeks later (Dunn and Wong, 2012). As those dendrites grow out, the different cell types adopt distinct strategies: some appear to target directly their nearby cone photoreceptor afferents, which in the mouse retina have all differentiated their stratifying cone pedicles in the OPL well before bipolar cell differentiation (Reese et al., 2005; Morgan et al., 2006); still others exhibit a more diffuse exploratory behavior, ultimately contacting an excess of cone pedicles before retracting to achieve their final dendritic structure (Lee et al., 2011; Dunn and Wong, 2012). That the presence of the cones is critical for achieving characteristic dendritic morphology is shown by eliminating the population of cone afferents before dendritic differentiation: while the overall extent of the dendritic field is unchanged, the patterned distribution of dendritic endings within the field area is lost entirely (Keeley and Reese, 2010). This does not appear to require transmission of neural activity at the cone-to-bipolar cell synapse for the Type 7 cell (Lee et al., 2011), yet visual activity has been shown to modulate the number of cones contacted for certain other CBC types (Dunn and Wong, 2012).

The unaltered areal extent of bipolar cells lacking their normal afferents indicates that the extent of dendritic outgrowth does not require the presence of cone pedicles. Rather, it suggests that this outgrowth is constrained by the presence of other neighboring homotypic bipolar cells, as each bipolar cell type is believed to differentiate a tiling dendritic arbor, extending to the limits of a neighboring cell’s dendritic field (Wässle et al., 2009). Genetic manipulations that either increase or decrease the density of homotypic neighbors yield dendritic arbors in maturity that are correspondingly smaller or larger, respectively (Lee et al., 2011). Furthermore, the number of cone pedicles contacted is also correspondingly reduced or increased, indicating that this cone-to-bipolar cell convergence ratio is not intrinsically specified (Lee et al., 2011).

Yet the fact that bipolar cells in a cone-depleted retina still acquire features of their mature morphology, including the correct laminar positioning of their dendrites in the OPL and their axonal stratification in the IPL, would suggest that cell-intrinsic factors play some role in the acquisition of their characteristic morphology (Keeley and Reese, 2010). With the advent of single cell transcriptional profiling, we now have the genetic signature discriminating each type of bipolar cell, and while many express common genes likely to be instrumental in the acquisition of their morphology, other genes are revealed to be relatively selectively expressed (Shekhar et al., 2016). These include transcription factors that may prove critical in the ultimate differentiation of the cell, including axonal stratification (e.g., Star et al., 2012), activating unique effector genes that control, for instance, the expression of cell adhesion molecules critical for achieving that stratification (e.g., Duan et al., 2014).

**Figure 9:**
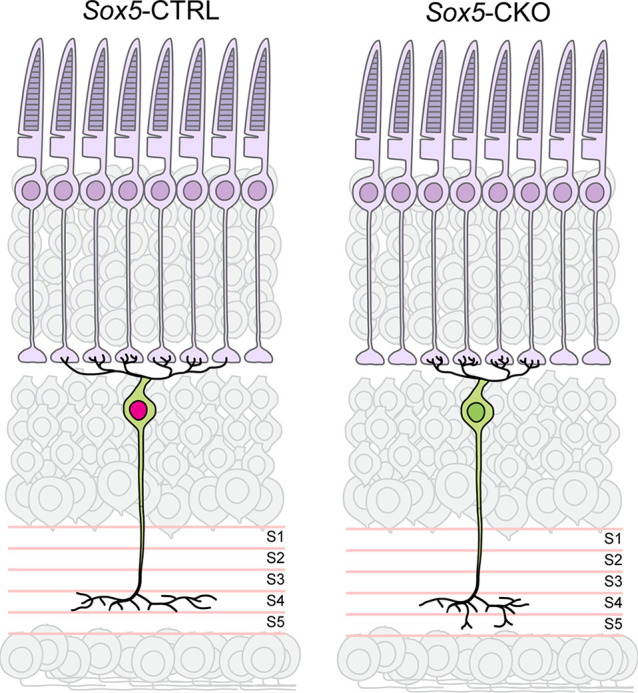
Summary diagram depicting the reduced size of axonal and dendritic arbors in the *Sox5*-CKO retina, indicative of a role for *Sox5* in promoting process outgrowth in the plane of the IPL and OPL, respectively. *Sox5* also contributes specificity to the targeting of those axonal arbors within S4 of the IPL, made apparent by the ectopic extension into S5 in its absence. The hyper-innervation of pedicles by dendritic branches is interpreted to arise secondarily, from the lack of shared pedicle contacts with neighboring Type 7 dendritic arbors, inferred from the reduction in dendritic field area by a population that normally tiles the retina.

The present study has demonstrated a role for *Sox5* in the differentiation of Type 7 CBCs. Relatively little is known about the role of *Sox5* in neural development. It has previously been shown to participate in the differentiation of deep cortical layer neurons in mice (Kwan et al., 2008), and loss of its orthologue in *Drosophila* results in a reduction in terminal bouton number in motor neurons, as well as a reduction in the size and complexity of the dendritic arbors of DA neurons (Li et al., 2017). Furthermore, it has been shown to function as a transcriptional activator of the cytoskeletal regulator, *Crmp5*, modulating the differentiation of hippocampal cells *in vitro* (Naudet et al., 2018). Here we have shown that bipolar cells lacking *Sox5* develop the characteristic morphological features of Type 7 cells, yet their axonal and dendritic field areas are reduced in size, while the axonal arbors exhibit sprouting of vertically oriented processes that expand their overall depth within the IPL, and their dendritic arbors hyper-innervate a smaller-than-normal number of cone pedicles (Figure 9). The functional consequence would yield smaller receptive fields though exhibiting enhanced average fidelity of signal transfer at each pedicle, transmitted to a broader range of ganglion cell targets in the IPL.

Normal Type 7 cone bipolar cells are believed to extend their axonal and dendritic territories to approximate a tiling of the retinal surface (Wässle et al., 2009; Helmstaedter et al., 2013). Developing Type 7 CBC dendritic arbors commence their morphological differentiation after the 1st postnatal week (Morgan et al., 2006; West et al., 2022), over-extending into the fields of their homotypic neighbors by the end of the 2nd postnatal week, and then subsequently retracting their dendrites and reducing the total number of pedicles initially contacted (Lee et al., 2011; Dunn and Wong, 2012). Analyses of dendritic structure at P18 show Type 7 CBC dendritic arbors in *Sox5*-CKO retinas that overlie more pedicles than seen for adult cells, also seen in control retinas, indicating that dendritic remodeling is still occurring at this age, and is not dependent on SOX5. However, cells in the in *Sox5-CKO* at P18 already exhibit smaller dendritic areas and overlie fewer cone pedicles relative to cells from their age-matched littermate controls, each being reduced by ~35% (data not shown), suggesting that the deficit in the CKO is due to compromised outgrowth rather than one of excessive retraction.

Having ultimately colonized a smaller domain that contains fewer cone pedicles due to the loss of this outgrowth-promoting function of *Sox5*, many of these Type 7 CBCs make more contacts per pedicle, on average, than they would otherwise have made. Occasional pedicles have been reported to connect to the dendritic arbors of two neighboring Type 7 cells at the borders of adjacent dendritic fields (Wässle et al., 2009; but see Behrens et al., 2016). As the number of dendritic contacts with a pedicle made by a Type 7 cell tends to decline with distance from the soma, shared pedicles presumably receive a reduced number of dendritic terminals from each of the two neighboring cells. The fact that the most hyper-innervated pedicles reside closer to the soma in the *Sox5*-CKO retinas (Figure 8F) is not at odds with this, because bipolar cells in the mouse retina appear to be arranged upon the retina no better than a random distribution of cells (Keeley et al., 2017, 2020), and dually innervated pedicles are occasionally present nearby individual Type 7 somata (Wässle et al., 2009). The increase in the average number of dendritic terminals per pedicle in the *Sox5*-CKO retina may, therefore, reflect a loss of such dually-contacted pedicles now being colonized by only one Type 7 cell, still leaving no pedicles free of contact and thus maintaining complete functional coverage of the retina.

The genes regulated by *Sox5* controlling these features of the Type 7 CBC morphology remain to be determined. The cadherin family of cell adhesion molecules is a well-studied group of proteins that helps mediate many aspects of neuronal differentiation, including dendritic and axonal outgrowth (Suzuki and Takeichi, 2008). Cadherins are also required for the wiring of specific circuits based on the selective expression of different family members within individual cell types (Sanes and Zipursky, 2020). The direction selective circuit in the mouse retina is one such example as Type 5 CBCs, which express cadherin 9 (*Cdh9*), stratify their axonal arbors in S4 where they make contacts with cholinergic amacrine cells and direction selective retinal ganglion cells; studies have shown that this cadherin is necessary for proper axonal stratification and connectivity within this circuit (Duan et al., 2014, 2018). While those studies suggested that *Cdh9* is exclusive to the Type 5 CBCs, single cell sequencing data indicates that the Type 7 CBCs, which also stratify in S4 and contact the same amacrine cell (Helmstaedter et al., 2013), express *Cdh9* as well (Shekhar et al., 2016); moreover, there is a predicted SOX5 binding site directly upstream of *Cdh9* in the putative promoter region, suggesting that SOX5 might directly regulate the expression of this cadherin in Type 7 cells. Future studies will be necessary to determine if *Cdh9* mediates SOX5-dependent process outgrowth, stratification, and circuit development, or if other downstream effectors of this transcription factor are required to account for the distinct roles identified here for the normal differentiation of the Type 7 cone bipolar cell.

## Data Availability Statement

The raw data supporting the conclusions of this article are available from the corresponding author upon request.

## Ethics Statement

All procedures were approved by the Institutional Animal Care and Use Committee at the University of California, Santa Barbara, and in accord with the NIH *Guide for the Care and Use of Laboratory Animals*.

## Author Contributions

BK, BR, and PK designed the experiments, interpreted the data, and wrote the manuscript. BK and PK performed the experiments. BK conducted the data analysis. All authors contributed to the article and approved the submitted version.

## Funding

This research was supported by grants from the National Institutes of Health (EY-019968 and OD-010610).

## Footnotes

^1^ singlecell.broadinstitute.org/single_cell

^2^ https://imagej.net/plugins/diffraction-psf-3d

^3^ https://imagej.net/plugins/iterative-deconvolve-3d
